# Characteristics and pathogenic role of adherent-invasive *Escherichia coli* in inflammatory bowel disease: Potential impact on clinical outcomes

**DOI:** 10.1371/journal.pone.0216165

**Published:** 2019-04-29

**Authors:** Jae Gon Lee, Dong Soo Han, Su Vin Jo, A. Reum Lee, Chan Hyuk Park, Chang Soo Eun, Yangsoon Lee

**Affiliations:** 1 Department of Internal Medicine, Hanyang University Guri Hospital, Hanyang University College of Medicine, Guri, Korea; 2 Department of Laboratory Medicine, Hanyang University Hospital, Hanyang University College of Medicine, Seoul, Korea; Kurume University School of Medicine, JAPAN

## Abstract

Adherent-invasive *Escherichia coli* (AIEC) has been reported as associated with the pathogenesis of inflammatory bowel disease (IBD). We aimed to investigate the characteristics of mucosa-associated *E*. *coli* and the clinical significance of AIEC in Korean IBD patients. *E*. *coli* strains were isolated from the mucosal tissues of 18 Crohn’s disease (CD) patients, 24 ulcerative colitis (UC) patients, and 9 healthy controls (HC). Adhesion, invasion, and survival assays were performed to evaluate phenotypic features of *E*. *coli* isolates and to identify AIEC. The presence of virulence genes and cytokine expression were examined using PCR. In addition, data on IBD-related hospitalization were collected. A total of 59 *E*. *coli* strains were isolated (25 from CD, 27 from UC, and 7 from HC). The average levels of adhesion, invasion, and survival were higher in *E*. *coli* strains from IBD patients than those from HC (adhesion: 1.65 vs. 0.71, *p* = 0.046; invasion: 1.68 vs. 0.52, *p* = 0.039; survival: 519.55 vs. 47.55, *p* = 0.363). Prevalence of AIEC in HC, CD and UC patients was 22.2%, 38.9% and 37.5%, respectively. *E*. *coli* isolates from IBD patients had various virulence genes and were associated with increased expression of TNF-α and IL-17. IBD-related hospitalization within 3 years was 18.8% in patients with AIEC and 11.5% in patients without AIEC. *E*. *coli* strains from IBD patients showed high levels of adhesion, invasion, and survival. AIEC strains were identified in both CD and UC patients at a similar rate. AIEC may be associated with sustaining inflammation in the pre-existing inflammatory mucosa.

## Introduction

Inflammatory bowel disease (IBD), including Crohn’s disease (CD) and ulcerative colitis (UC), is characterized by chronic inflammation of the gastrointestinal tract. Although the etiology of IBD is not fully understood, it is generally accepted that genetic susceptibility, environmental factors, and altered intestinal microbiota are involved [1]. A dysbiosis, characterized by an increase in the number of mucosa-associated colitogenic bacteria and a reduction in overall biodiversity, has been suggested as an important contributor to the pathogenesis of IBD [2]. Previous studies have reported that increased numbers of mucosa-associated *Escherichia coli* strains were observed in patients with IBD [3, 4]. Adherent-invasive *E*. *coli* (AIEC), in particular, has been proposed as a possible pathogen that may potentially induce intestinal inflammation [5].

Several factors have been identified for the interaction between AIEC and the intestinal mucosa in IBD. AIEC strains are able to adhere to intestinal epithelial cells (IECs) using type 1 pili that bind to carcinoembryonic antigen-related cell-adhesion molecule 6 (CEACAM6) receptors on enterocytes, which are overexpressed on the surface of IECs in patients with CD [6]. In addition, AIEC is able to invade into the lamina propria and Peyer’s patches through M cells via long polar fimbriae [7]. AIEC not only can be internalized into macrophages, but also can survive and replicate within macrophages due to host autophagy defect. Then, AIEC induces the release of tumor necrosis factor alpha (TNF-α) by the activation of infected macrophages, and also induces Th17 and CD8+ cytotoxic responses [5, 8].

Thus, AIEC strains are considered to play an important role in the intestinal inflammatory responses. To date, most studies on AIEC have been conducted in Western countries, and inconsistent data exist regarding the prevalence and pathogenic role of AIEC in patients with IBD [1]. Therefore, we aimed to investigate the characteristics of mucosa-associated *E*. *coli*, identify AIEC, and evaluate the clinical significance of AIEC in Korean IBD patients.

## Materials and methods

### Study subjects

9 healthy controls (HCs), 18 CD patients, and 24 UC patients underwent colonoscopy and biopsy. Subjects signed an informed consent prior to undergoing endoscopy. HCs underwent colonoscopy for other reasons such as cancer screening, and tissues were taken from the ileocecal valve or rectum. In patients with IBD, tissues were taken from the inflamed areas for patients with active disease and from the ileocecal valve or rectum for patients with inactive disease. Disease activity was assessed endoscopically, using the simple endoscopic score for Crohn’s disease (SES-CD) [9] and ulcerative colitis endoscopic index of severity (UCEIS) [10]. SES-CD <3 and UCEIS ≤1 were considered endoscopically inactive disease. We reviewed medical records to obtain demographic characteristics, disease location and behavior, prior therapy, and IBD-related hospitalization rates. All samples were stored at -80°C until further analysis. The study protocol was approved by the Institutional Review Board of Hanyang University Guri Hospital (IRB No. 2014-10-011-001).

### *E*. *coli* strains

Each biopsy sample was washed with phosphate-buffered saline (PBS) and homogenized using a pestle. Next, tissue lysates were diluted and plated onto MacConkey agar plates to obtain colonies. *E*. *coli* strains were identified using the matrix assisted laser desorption/ionization time-of-flight (MALDI-TOF) mass spectrometry system from Bruker Biotyper (Bruker Daltonics, Bremen, Germany). All *E*. *coli* strains were phylotyped into A, B1, B2, and D groups using a multiplex polymerase chain reaction (PCR) method. Reference *E*. *coli* strains K-12 and LF82 were used as a negative and positive control, respectively. When ≥2 strains were isolated from one subject, Enterobacterial Repetitive Intergenic Consensus (ERIC)-PCR was performed to evaluate clonality as previously described by Ardakani et al. [11].

### Adhesion assays

HEp-2 cell monolayers were cultured in 12-well plates at a density of 1 x 10^5^ cells/well at 37°C for 24 h, infected with 1 ml bacterial suspension (10^7^ bacteria/ml; multiplicity of infection [MOI] = 100), and incubated at 37°C for 3 h [12]. After incubation, infected cell monolayers were washed with PBS, fixed in methanol and Giemsa-stained. Then cell layers were examined using a light microscope. Adhesion index, defined as the mean number of bacteria per cell after examination of 20 visual fields in three independent experiments, was measured for each isolate. Positive adhesion was defined when the adhesion index was ≥1. Grade of adhesion was defined as follows: grade 1, adhesion index ≥1; grade 2, adhesion index ≥10; grade 3, adhesion index ≥100 [13].

### Invasion assays

The ability of all *E*. *coli* isolates to invade host cells was examined using the gentamicin protection assay, described by Darfeuille-Michaud et al. [14]. HEp-2 cells were seeded in 24-well tissue culture plates at a density of 1 x 10^5^ cells/well, infected with 0.5 ml bacterial suspension (5 x 10^6^ bacteria/ml; MOI = 50), and incubated at 37°C for 3 h. After incubation, infected cell monolayers were washed four times with PBS, supplemented with fresh medium containing 100 μg/ml gentamicin (Sigma) to kill extracellular bacteria, and further incubated for 1 h. Then cells were washed and lysed with a 1% Triton X-100 (Sigma) solution, and cell lysates dilutions were plated on MacConkey agar. Invasion level, defined as the percentage of intracellular bacteria compared with the initial inoculum, was measured. An isolate was considered invasive when the invasion level was ≥0.1%.

### Survival assays

THP-1 cells were seeded in 24-well tissue culture plates at a density of 1 x 10^5^ cells/well and infected with 0.1 ml bacterial suspension (1 x 10^6^ bacteria/ml; MOI = 10). Bacteria were centrifuged onto cell monolayers at 500 x *g* for 10 min and incubated at 37°C for 10 min. After incubation, infected macrophages were washed twice with PBS and supplemented with fresh medium containing 100 μg/ml gentamicin to kill extracellular bacteria. After a 1 h of additional incubation period, the medium was removed and fresh medium with 50 μg/ml gentamicin was added for either 1 or 24 h. Then, cells were washed and lysed with a 1% Triton X-100 solution, and cell lysates dilutions were plated on MacConkey agar. Survival level, defined as the ratio between the number of intracellular bacteria recovered after 24 h of incubation and those recovered after 1 h, was measured. An isolate was considered to have survival capability when the survival level was ≥100% [14]. All assays were performed three times in separate experiments, and details of the used cell lines are shown in S1 Table.

### Determination of AIEC

AIEC strains were determined when all of the following three conditions were met: (1) adhesiveness, adhesion index ≥1; (2) invasiveness, invasion level ≥0.1%; and (3) survival and replication, survival level ≥100% [1].

### Virulence genotyping

The presence of 21 virulence factors (*fimH*, *afa-dra*, *sfa-foc*, *hra*, *eaeA*, *ibeA*, *tia*, *ipaH*, *fyuA*, *chuA*, *kpsMT* II, *kps MT* I*(K1)*, *hlyA*, *estA*, *estB*, *ompA*, *pic*, *aggR*, and *yjaA*) was examined by PCR using appropriate primers. A detailed procedure for the identification of each virulence factor was performed with reference to a previous report [15].

In addition, to evaluate the genetic polymorphism of *E*. *coli* isolates, *fimH* gene was amplified by PCR and sequenced for identification of hotspot mutations reported in a previous study [16]. We searched for three points of mutation (G73A/E/R/W, T158A/P, R166C/H/S) in the amino acid sequence ranging from 15AA to 289AA.

### Reverse transcription PCR (RT-PCR)

Total RNA was extracted from cell lines infected with *E*. *coli* isolates and controls using a Hybrid-R Total RNA Isolation Kit (GeneAll Biotechnology, Seoul, Korea), and quantified using a Biospec-nano spectrophotometer (Life Science, Columbia, MD, USA). The PCR products were resolved by electrophoresis on 1.5% agarose gels containing ethidium bromide, and bands were visualized using a ChemiDoc XRS+ System (Bio-Rad, CA, USA). The primer sequences used for PCR are shown in S2 Table.

### Statistical analysis

Continuous and categorical variables among groups were compared using the Mann-Whitney test and the chi-square or Fisher’s exact test, respectively. A *p* value of <0.05 was considered statistically significant. All statistical procedures were conducted using IBM SPSS Statistics 20.0 (IBM Corp., Armonk, NY, USA).

## Results

### Characteristics of the study subjects

Table 1 shows the baseline characteristics of the study subjects. *E*. *coli* strains were isolated in 6 of 9 HC subjects, 16 of 18 CD patients, and 19 of 24 UC patients. A total of 83.3% of CD patients had ileocolonic disease, and 61.1% had non-stricturing and non-penetrating disease, while 54.2% of UC patients had pancolitis. 72.2% of CD patients and 29.2% of UC patients had been exposed to thiopurines, and 33.3% of CD patients had been exposed to anti-TNF agent.

**Table 1:** Characteristics of the study subjects.

Variables		HC (n = 9)	CD (n = 18)	UC (n = 24)
Age		55.0 (37.0–57.0)	25.0 (22.0–27.0)	36.0 (26.0–46.0)
Sex	Male	4 (44.4)	11 (61.1)	10 (41.7)
	Female	5 (55.6)	7 (38.9)	14 (58.3)
Biopsy site	Ileum	0 (0)	5 (27.8)	0 (0)
	Ileocecal valve	0 (0)	7 (38.9)	3 (12.5)
	Colon	9 (100)	6 (33.3)	21 (87.5)
Number of subjects positive for *E*. *coli*		6 (66.7)	16 (88.9)	19 (79.2)
CD location*	Ileal	-	1 (5.6)	-
	Colonic	-	2 (11.1)	-
	Ileocolonic	-	15 (83.3)	-
	Isolated upper disease	-	0 (0)	-
CD behavior	Non-stricturing and non-penetrating	-	11 (61.1)	-
	Stricturing	-	3 (16.7)	-
	Penetrating	-	4 (22.2)	-
UC extent*	Proctitis	-	-	5 (20.8)
	Left-sided colitis	-	-	6 (25.0)
	Pancolitis	-	-	13 (54.2)
Disease activity^†^	Active	-	14 (77.8)	15 (62.5)
	Inactive	-	4 (22.2)	9 (37.5)
Treatment exposure	Aminosalicylates	-	12 (66.7)	15 (62.5)
	Steroids	-	10 (55.6)	3 (12.5)
	Thiopurine	-	13 (72.2)	7 (29.2)
	Anti-TNF agent	-	6 (33.3)	0 (0)
	Methotrexate	-	1 (5.6)	0 (0)

HC, healthy control; CD, Crohn’s disease; UC, ulcerative colitis. Values are given as median (interquartile range) or numbers (%).

*Present location/extent when active disease, previous location/extent when inactive disease.

^†^Simple endoscopic score for Crohn’s disease (SES-CD) and ulcerative colitis endoscopic index of severity (UCEIS) were used to assess the endoscopic activity. SES-CD <3 and UCEIS ≤1 were considered endoscopically inactive diseases.

### Characteristics of the isolated *E*. *coli* strains

**Fig 1:**
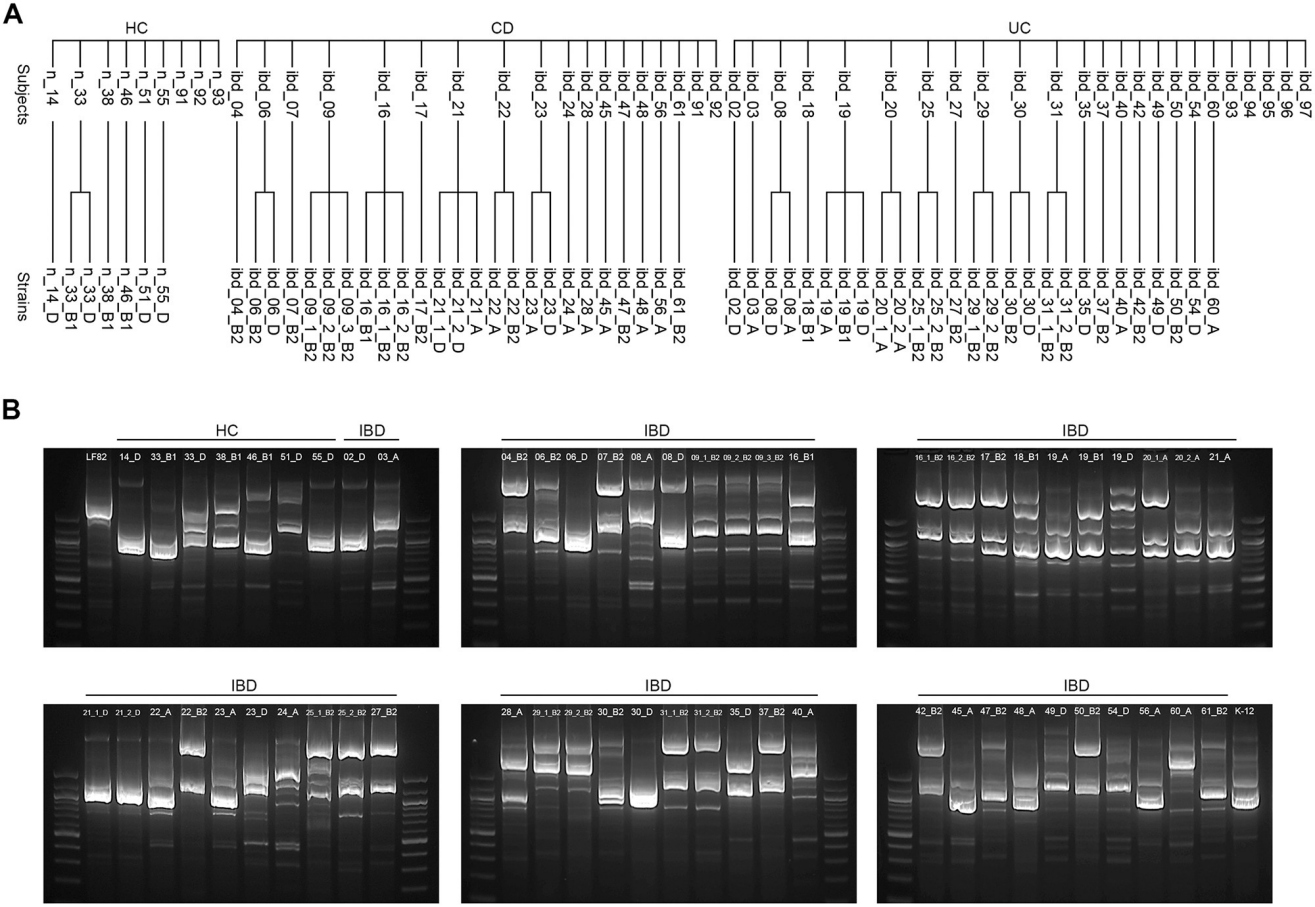
Classification of *E*. *coli* isolates. (A) Isolated *E*. *coli* strains from individual study subjects. (B) ERIC-PCR results. Prefex "n_" and "ibd_" represent healthy control and inflammatory bowel disease patients, respectively. Suffix "_A", "_B1", "_B2", and "_D" represent phylotypes. HC, healthy control; CD, Crohn’s disease; UC, ulcerative colitis.

A total of 59 *E*. *coli* strains were isolated from 41 subjects: 7 strains from 6 HC subjects, 25 from 16 CD patients, and 27 from 19 UC patients. There were cases in which 2–3 strains were isolated from one subject. ERIC-PCR results demonstrated that the strains belonging to the same phylogroup showed similar electrophoresis patterns, suggesting that these strains are clonal. In contrast, the strains belonging to the different phylogroups showed distinct electrophoresis patterns, suggesting that these strains are not clonal (Fig 1).

Table 2 shows phylogenetic groups of *E*. *coli* isolates. Phylotype B2 made up 44.2% of *E*. *coli* strains in IBD patients, as compared with 0% in HC.

**Table 2:** *E*. *coli* isolate phylogenetic groups.

Phylotype	HC (n = 7)	CD (n = 25)	UC (n = 27)	Total (n = 59)
A	0 (0)	8 (32.0)	7 (25.9)	15 (25.4)
B1	3 (42.9)	1 (4.0)	2 (7.4)	6 (10.2)
B2	0 (0)	12 (48.0)	11 (40.7)	23 (39.0)
D	4 (57.1)	4 (16.0)	7 (25.9)	15 (25.4)

HC, healthy control; CD, Crohn’s disease; UC, ulcerative colitis. Values are given as the number (%).

*E*. *coli* strains from IBD patients showed significantly higher adhesion grade than those from HC subjects (1.65 ± 0.99 vs. 0.71 ± 0.49, *p* = 0.046). There were no significant differences in grade of adhesion according to phylotype and the presence of inflammation.

Although it was not statistically significant, the invasiveness of *E*. *coli* strains isolated from IBD patients was higher than those isolated from HC subjects (1.68 ± 0.27 vs. 0.52 ± 0.15, *p* = 0.055). Among them, *E*. *coli* strains from UC patients showed significantly higher invasion level than those from HC subjects (2.03 ± 0.34 vs. 0.52 ± 0.15, *p* = 0.032), and those from CD patients (2.03 ± 0.34 vs. 1.33 ± 0.42, *p* = 0.047). Phylotype A and B1 showed significantly higher invasion level than other phylotypes (*p*<0.001). There was no significant difference in invasion level according to the presence of inflammation.

*E*. *coli* strains from IBD patients showed higher average survival level than those from HC subjects, but it was not statistically significant (519.55 ± 130.82 vs. 47.55 ± 15.74, *p* = 0.363). Phylotype A showed significantly higher survival level than other phylotypes (*p* = 0.002), and *E*. *coli* strains isolated from inflamed tissues showed significantly higher survival level than those from non-inflamed tissues (628.73 ± 177.16 vs. 289.99 ± 157.98, *p* = 0.001).

*E*. *coli* strains from CD patients with stricturing or penetrating behavior showed increased grade of adhesion and survival level than those from CD patients without stricturing or penetrating behavior, but it was not statistically significant (grade of adhesion: 2.0 ± 0.29 vs. 1.31 ± 0.20, *p* = 0.074; survival level: 969.20 ± 545.20 vs. 338.0 ± 158.11, *p* = 0.061) (Table 3).

**Table 3:** Characteristics of the isolated *E*. *coli* strains.

Characteristics		Grade of adhesion^a^	*p* value	Invasion level^b^	*p* value	Survival level^c^	*p* value
Reference strains	K-12	0.05 ± 0.04	-	0.05 ± 0.02	-	2.61 ± 1.83	-
	LF82	1.05 ± 0.58		1.05 ± 0.16		116.07 ± 22.07	
Disease group	HC (n = 7)	0.71 ± 0.49	0.046	0.52 ± 0.15	0.039	47.55 ± 15.74	0.363
	CD (n = 25)	1.56 ± 0.87		1.33 ± 0.42		634.10 ± 244.00	
	UC (n = 27)	1.74 ± 1.10		2.03 ± 0.34		420.86 ± 123.84	
	IBD (n = 52)	1.65 ± 0.99		1.68 ± 0.27		519.55 ± 130.82	
Phylotype	A (n = 15)	2.07 ± 0.03	0.064	1.88 ± 0.37	<0.001	964.73 ± 374.76	0.002
	B1 (n = 6)	1.67 ± 0.42		4.16 ± 1.40		543.84 ± 294.28	
	B2 (n = 23)	1.39 ± 0.19		0.93 ± 0.26		229.69 ± 125.62	
	D (n = 15)	1.20 ± 0.28		0.70 ± 0.21		290.99 ± 108.00	
Inflammation	Absent (n = 18)	1.72 ± 0.27	0.412	2.63 ± 0.66	0.105	289.99 ± 157.98	0.001
	Present (n = 34)	1.62 ± 0.16		1.18 ± 0.21		628.73 ± 177.16	
CD behavior	B1 (n = 16)	1.31 ± 0.20	0.074	1.62 ± 0.67	0.450	338.00 ± 158.11	0.061
	B2/B3 (n = 9)	2.00 ± 0.29		0.89 ± 0.22		969.20 ± 545.20	

HC, healthy control; CD, Crohn’s disease; UC, ulcerative colitis; IBD, inflammatory bowel disease; B1, nonstricturing and nonpenetrating; B2, stricturing; B3, penetrating. Values are given as the mean ± standard error of three independent experiments.

^a^1, adhesion index ≥1; 2, adhesion index ≥10; 3, adhesion index ≥100

^b^Percentage of inoculum surviving after 1 h of gentamicin treatment (number of intracellular bacteria / initial inoculum x 100)

^c^Number of intracellular bacteria at 24 h post-infection / number of bacteria at 1h post-infection x 100 (%)

### Identification of AIEC

AIEC strains were identified in 2 of 7 strains isolated from HC subjects (28.6%), 7 of 25 from CD patients (28.0%), and 12 of 27 from UC patients (44.4%) based on the criteria used to define AIEC strains as described in the methods. Among the 21 AIEC strains, 9 (42.9%), 2 (9.5%), 5 (23.8%), and 5 (23.8%) were identified as phylotype A, B1, B2, and D, respectively. Prevalence of AIEC was 22.2% (2 of 9) in HC subjects, 38.9% (7 of 18) in CD patients, and 37.5% (9 of 24) in UC patients. There were no significant differences in the prevalence of AIEC according to disease activity and treatment exposure (Table 4).

**Table 4:** Prevalence of adherent-invasive *E*. *coli*.

Characteristics		Number of subjects positive for AIEC (%)	*p* value
Disease group	HC (n = 9)	2 (22.2)	0.407
	CD (n = 18)	7 (38.9)	
	UC (n = 24)	9 (37.5)	
Disease activity	Active IBD (n = 29)	11 (37.9)	0.618
	Inactive IBD (n = 13)	5 (38.5)	
Treatment exposure	Anti-TNF naïve (n = 36)	14 (38.9)	0.587
	Anti-TNF experienced (n = 6)	2 (33.3)	
	Thiopurine naïve (n = 22)	9 (40.9)	0.470
	Thiopurine experienced (n = 20)	7 (35.0)	

AIEC, adherent-invasive *E*. *coli*; HC, healthy control; CD, Crohn’s disease; UC, ulcerative colitis; IBD, inflammatory bowel disease

### Virulence genotyping

Various genes associated with virulence factors were identified. The *fimH* gene was identified in 100% of both control and IBD patients. Prevalence of *yjaA* gene was significantly higher in *E*.*coli* isolates from CD patients than those from UC patients and HC. Although it was not statistically significant, several virulence genes such as *ibeA*, *ipaH*, *fyuA*, *kpsMT* II, and *K1* were more common in strains from IBD patients than those from HC (Table 5).

**Table 5:** Virulence genotyping.

Function	Genes	Number of isolates with virulence genes (%)			*p* value
		HC (n = 7)	CD (n = 25)	UC (n = 27)	
Adhesin	*fimH*	7 (100)	25 (100)	27 (100)	-
	*afa-dra*	6 (85.7)	21 (84.0)	22 (81.5)	0.952
	*sfa-foc*	4 (57.1)	11 (44.0)	16 (59.3)	0.527
	*hra*	1 (14.3)	3 (12.0)	5 (18.5)	0.806
	*eaeA*	3 (42.9)	11 (44.0)	13 (48.1)	0.943
Invasin	*ibeA*	0 (0.0)	4 (16.0)	4 (14.8)	0.532
	*tia*	6 (86.0)	19 (76.0)	21 (77.8)	0.860
	*ipaH*	2 (28.6)	15 (60.0)	15 (55.6)	0.331
Siderophore	*fyuA*	4 (57.1)	19 (76.0)	17 (63.0)	0.491
	*chuA*	4 (57.1)	16 (64.0)	18 (66.7)	0.894
Capsule	*kpsMT* II	2 (28.6)	16 (64.0)	14 (51.9)	0.237
	*kpsMT* I (*K1*)	1 (14.3)	9 (36.0)	6 (22.2)	0.385
Toxin	*hlyA*	7 (100)	23 (92.0)	26 (96.3)	0.631
	*estA*	0 (0.0)	0 (0.0)	0 (0.0)	-
	*estB*	2 (28.6)	5 (20.0)	3 (11.1)	0.475
Protectin	*ompA*	7 (100)	25 (100)	27 (100)	-
Miscellaneous	*pic*	7 (100)	23 (92)	23 (85.2)	0.459
	*aggR*	5 (71.4)	22 (88.0)	25 (92.6)	0.304
	*yjaA*	0 (0.0)	19 (76.0)	13 (48.1)	0.001
	*tsp*	5 (71.4)	14 (56.0)	17 (63.0)	0.731

HC, healthy control; CD, Crohn’s disease; UC, ulcerative colitis

In addition, mutation of the *fimH* gene was analyzed. Sequencing results of the *fimH* gene were obtained from 52 out of 59 isolated strains. As a result, R166H mutation was identified in three AIEC strains (ibd_07_B2 strain and ibd_28_A strain from CD patients, ibd_25_2_B2 strain from UC patient). All three patients had active disease. In contrast, no hotspot mutations were identified in non-AIEC strains (Table 6).

**Table 6:** Mutations of the *fimH* gene in *E*. *coli* isolates.

Strain	Group	Phenotype	Amino acid										
			25	27	70	78	104	119	150	162	163	166	227
K-12	Reference	non-AIEC	A	V	N	S	P	A	A	D	V	R	R
LF82	Reference	AIEC		A	S	N							
n_38_B1	HC	AIEC		A									
n_51_D	HC	AIEC		A	S	N							
ibd_03_A	UC	AIEC		A									
ibd_06_B2	CD	AIEC					L						
ibd_06_D	CD	AIEC		A				V					S
ibd_07_B2	CD	AIEC		A								**H**	
ibd_19_B1	UC	AIEC		A									
ibd_22_B2	CD	AIEC		A	S								
ibd_25_1_B2	UC	AIEC		A	S	N					A		
ibd_25_2_B2	UC	AIEC		A								**H**	
ibd_28_A	CD	AIEC		A								**H**	
ibd_30_D	UC	AIEC											
ibd_31_1_B2	UC	AIEC		A									
ibd_31_2_B2	UC	AIEC		A									
ibd_40_A	UC	AIEC		A									
ibd_49_D	UC	AIEC		A	S	N							
ibd_54_D	UC	AIEC		A	S	N							
ibd_60_A	UC	AIEC		A									
n_14_D	HC	non-AIEC											
n_33_B1	HC	non-AIEC		A				V					
n_33_D	HC	non-AIEC		A				V					
n_46_B1	HC	non-AIEC		A									
n_55_D	HC	non-AIEC											
ibd_02_D	UC	non-AIEC											
ibd_04_B2	CD	non-AIEC			S	N							
ibd_08_A	UC	non-AIEC	T	A		N							
ibd_08_D	UC	non-AIEC											
ibd_09_1_B2	CD	non-AIEC					L						
ibd_09_2_B2	CD	non-AIEC					L						
ibd_09_3_B2	CD	non-AIEC					L						
ibd_16_B1	CD	non-AIEC		A									
ibd_16_1_B2	CD	non-AIEC		A									
ibd_16_2_B2	CD	non-AIEC		A									
ibd_17_B2	CD	non-AIEC		A		N							
ibd_18_B1	UC	non-AIEC		A									
ibd_19_D	UC	non-AIEC		A					S				
ibd_20_1_A	UC	non-AIEC		A		N							
ibd_21_1_D	CD	non-AIEC											
ibd_21_2_D	CD	non-AIEC											
ibd_23_D	CD	non-AIEC		A	S	N							
ibd_27_B2	UC	non-AIEC		A									
ibd_29_1_B2	UC	non-AIEC		A	S								
ibd_29_2_B2	UC	non-AIEC		A	S								
ibd_30_B2	UC	non-AIEC					L						
ibd_35_D	UC	non-AIEC		A		N				V			
ibd_37_B2	UC	non-AIEC			S	N							
ibd_42_B2	UC	non-AIEC		A									
ibd_47_B2	CD	non-AIEC					L						
ibd_48_A	CD	non-AIEC		A									
ibd_50_B2	UC	non-AIEC											
ibd_56_A	CD	non-AIEC											
ibd_61_B2	CD	non-AIEC					L						

Blanks indicate identical to wild type, and only the mutations are shown. Prefex "n_" and "ibd_" represent healthy control and inflammatory bowel disease patients, respectively. Suffix "_A", "_B1", "_B2", and "_D" represent phylotypes. HC, healthy control; CD, Crohn’s disease; UC, ulcerative colitis; AIEC, adherent-invasive *E*. *coli*

### Expression of cytokines

**Fig 2:**
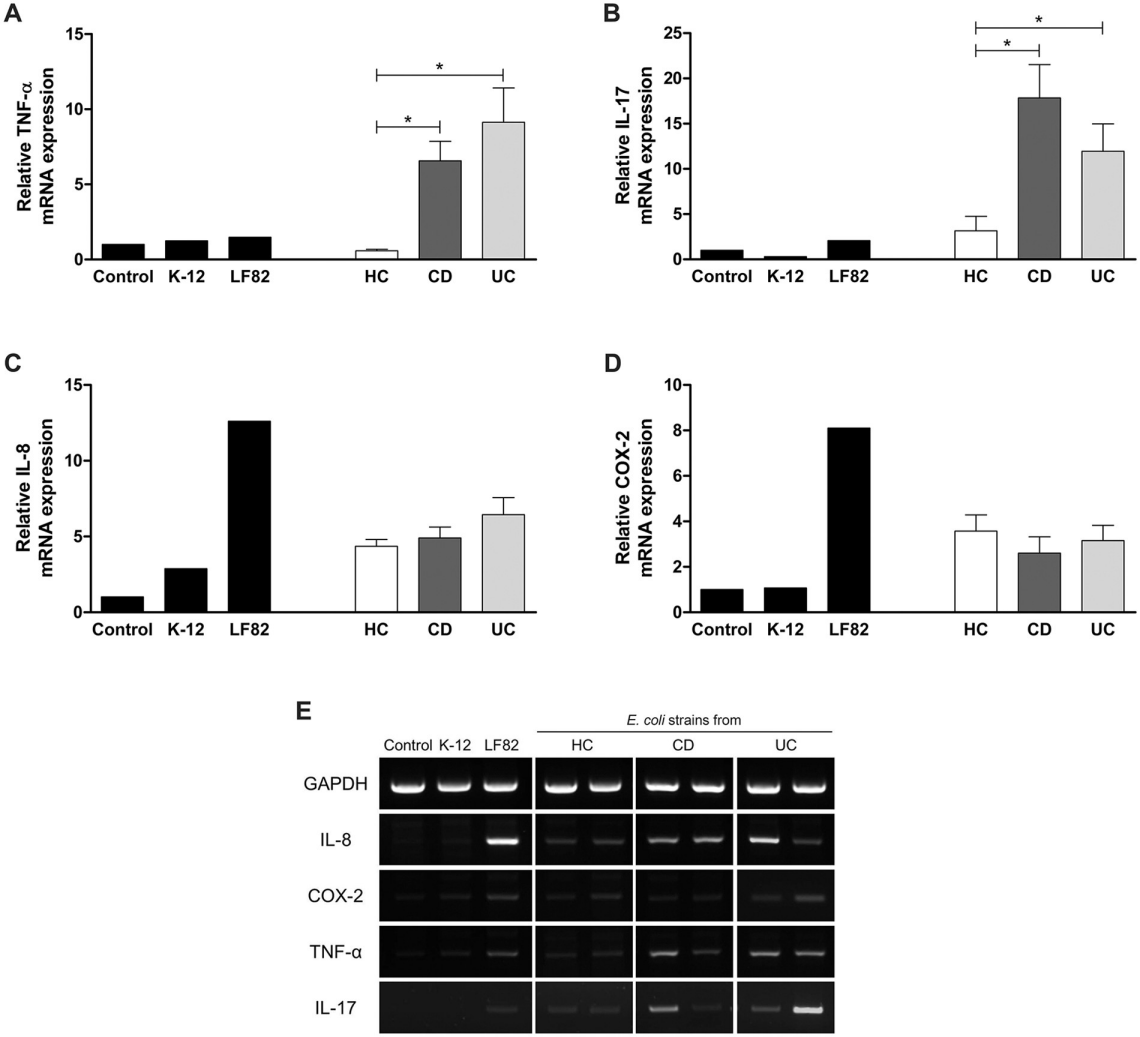
RT-PCR analyses for the expression of cytokines in *E*. *coli*-infected cell lines. The expression of TNF-α (A) and IL-17 (B) was significantly increased by *E*. *coli* strains from IBD patients, while the expression of IL-8 (C) and COX-2 (D) was not increased. HC, healthy control; CD, Crohn’s disease; UC, ulcerative colitis; TNF-α, tumor necrosis factor alpha; IL, interleukin; COX-2, cyclooxygenase 2 *Statistically significant (*p*<0.05).

The expression of TNF-α and interleukin 17 (IL-17) was significantly increased in HEp-2 cells infected with *E*. *coli* isolates from IBD patients than those from HC subjects, whereas the expression of cyclooxygenase 2 (COX-2) and IL-8 was not significantly different between groups (Fig 2). In Caco-2 cells infected with *E*. *coli* isolates with survival capability, the relative mRNA expression level of CEACAM6 was increased by two-fold over baseline.

### Clinical impact of AIEC

To determine the potential impact of AIEC on clinical outcomes, we assessed IBD-related hospitalization rates within 3 years according to the presence of AIEC at baseline. A total of 42 IBD patients (18 patients with CD, 24 with UC) were followed up for a median of 22.3 months (interquartile range, 14.3 months), and the causes of hospitalization included disease flares, IBD complications and surgery. IBD patients with AIEC showed a higher rate of IBD-related hospitalization than those without AIEC, but it was not statistically significant (3 of 16 patients, 18.8% vs. 3 of 26 patients, 11.5%, *p* = 0.658).

## Discussion

In the present work, we evaluated characteristics of mucosa-associated *E*. *coli* and identified AIEC in Asian IBD patients. *E*. *coli* isolates from IBD patients showed higher adhesiveness, invasiveness, and survival capability than those from HC subjects. Prevalence of AIEC strains was relatively higher in IBD patients than HC subjects, and similar in CD and UC patients. AIEC strains from IBD patients were associated with increased expression of proinflammatory cytokines and increased rates of IBD-related hospitalization.

Phylotype B2 made up 44.2% of *E*. *coli* strains from IBD patients and together with phylotype A, made up the majority of strains. Previous studies reported that phylotype B2 was the most common among mucosa-associated *E*. *coli* isolates, and it was associated with inflammation [17, 18]. We observed phylotype B2 was the most common, but there were no significant increases in adhesion, invasion, and survival than other phylotypes.

Prevalence of AIEC in patients with IBD varies widely across studies in the West, ranging from 21.7% to 62.5% in CD patients [14, 19, 20] and from 0% to 10% in UC patients [14, 21, 22]. Little is known about the prevalence and pathogenic role of AIEC in UC patients. Although a few studies have reported a higher prevalence of adherent *E*. *coli* strains in UC patients, these studies did not investigate the presence of AIEC [23, 24]. In some previous studies, *E*. *coli* strains from UC patients were reported to be associated with severe disease activity, increased fecal calprotectin, and increased expression of pro-inflammatory cytokines [25, 26]. Our results showed that the prevalence of AIEC in UC patients was comparable to the prevalence in CD patients (37.5% vs. 38.9%). We also observed increased expression of TNF-α and IL-17. Interestingly, the average invasion level was higher in *E*. *coli* strains from UC patients than those from CD patients. In patients with CD, Darfeuille-Michaud et al. [14] reported that AIEC strains were found more frequently in the ileum than in the colon, whereas our results showed that AIEC strains were present not only in the ileum but also in the colon.

To our knowledge, this is the first study to identify AIEC in Asian IBD patients. As mentioned above, our results on the prevalence and distribution of AIEC differed from those have been reported in Western countries. These differences may be explained by the presence of various factors that determine AIEC colonization in the intestinal mucosa of IBD patients. The composition and distribution of the intestinal microbiota may vary according to disease phenotype and genotype in patients with IBD [27], and other host and/or environmental factors might be involved.

We found some virulence genes, including *yjaA*, *ibeA*, *ipaH*, *fyuA*, *kpsMT* II, and *K1*, were more common in *E*. *coli* strains from IBD patients than those from HC subjects. However, this does not mean that *E*. *coli* strains having these virulence factors are more pathogenic because *E*. *coli* can evolve according to the intestinal environment, and the expression of virulence genes can be increased by the intestinal inflammation [28]. It may be difficult to identify the cause-effect relationship between bacterial virulence genes and the pathogenesis of IBD.

The adherence of AIEC is dependent on type 1 pili expression on the bacterial surface and on CEACAM6 expression on the apical surface of IECs, which is increased in enterocytes of patients with CD [29]. In addition, AIEC isolates were reported to be able to induce intestinal inflammation by expressing CEACAM6 [30]. Our results showed the increased expression of CEACAM6 in cell lines infected with *E*. *coli* isolates with survival capability. In other words, the increased expression of CEACAM6 on enterocytes can increase the invasion of *E*. *coli*, while the intracellular infection of *E*. *coli* can increase the expression of CEACAM6. This implies that the interaction between enterocytes and microbiota plays an important role in inducing intestinal inflammation.

It is unclear whether AIEC strains trigger intestinal inflammation or whether they present as a consequence of inflammation in patients with IBD. AIEC strains are considered pathobionts because they are able to evolve in specific and susceptible hosts and promote intestinal inflammation by inducing the expression of proinflammatory cytokines [1, 5]. Our results showed that AIEC was present in the uninflamed mucosa as well as in the inflamed mucosa of IBD patients, which are consistent with previous findings [21]. There were no significant differences in adhesiveness and invasiveness of *E*. *coli* isolates according to the presence of inflammation, but the survival capability was significantly higher in *E*. *coli* isolates from the inflamed mucosa than those from the uninflamed mucosa. In addition, we found that *E*. *coli* strains were present in the normal mucosa of HC subjects. However, *E*. *coli* isolates from HC subjects did not increase the expression of TNF-α and IL-17, whereas those from IBD patients increased the expression of TNF-α and IL-17. Furthermore, although it was not statistically significant because of the small sample size, IBD patients with AIEC tended to have a higher rate of IBD-related hospitalization than those without AIEC. Taken together, these results suggest that AIEC is only pathogenic in a specific intestinal environment; AIEC may survive and induce further inflammation in the pre-existing inflammatory mucosa, whereas it may simply colonize without inducing inflammation in the uninflamed mucosa. AIEC may perpetuate chronic inflammation after inflammation has been evoked, leading to IBD-related complications and hospitalizations.

There are some limitations in this study. Because the number of HC subjects was relatively small, it was difficult to obtain statistical differences between IBD patients and HC subjects. Second, the presence of tissue inflammation was determined endoscopically, but not histologically. Thus, our results regarding characteristics of *E*. *coli* isolates according to the presence of inflammation may be inconclusive. Third, because we performed in vitro experiments with *E*. *coli* isolates only, it is not possible to understand the interactions between *E*. *coli* isolates and other abundant bacterial community, and the role of AIEC in the actual intestinal microenvironment. In addition, the abundance of *E*. *coli* as a whole was unable to confirm, because we did not count colonies after the tissue culture. Fourth, we could not assess the difference in *E*. *coli* phenotype according to patients’ genotype, because genomic data were not collected. Finally, MOI was adjusted for appropriate experimental results in our study, which may be an arbitrary measure. However, considering the previous study that there was no significant difference in the invasion assays tested with MOI = 10 and MOI = 100 [31], adjustment of MOI does not seem to be a significant drawback in interpreting the result.

Despite these limitations, this work will provide a better understanding of the possible role of mucosa-associated *E*. *coli* in the pathogenesis of IBD. *E*. *coli* isolates from IBD patients showed higher levels of adhesion, invasion, and survival than those from HC subjects. AIEC strains were identified from intestinal mucosal tissues of both CD and UC patients at a similar rate. AIEC may be associated with sustaining inflammation in the pre-existing inflammatory mucosa, leading to disease progression and related complications.

## Supporting information

S1 Table Cell lines used in this study. (PDF)

S2 Table Primers used in this study. (PDF)
