# Supplementary material for: Characteristics and pathogenic role of adherent-invasive Escherichia coli in inflammatory bowel disease: Potential impact on clinical outcomes
Source: PLoS One. 2019 Apr 29;14(4):e0216165. doi: 10.1371/journal.pone.0216165 (PMC6488085; doi:10.1371/journal.pone.0216165)
Supplement: S1 Table — (PDF) [file pone.0216165.s001.pdf]

S1 Table. Cell lines used in this study

Cell lines	Purpose	Maintaining medium
HEp-2 cells	Adhesion assay	Dulbecco's modified Eagle medium (DMEM; Hyclone, South Logan, UT, USA)
	Invasion assay	
	Cytokine expression	
Caco-2 cells	CEACAM6 expression	Minimum Essential Medium (Hyclone)
THP-1 cells	Survival assay	RPMI 1640 medium (Hyclone)

CEACAM6, carcinoembryonic antigen-related cell-adhesion molecule 6

All cell lines were supplemented with 10% heat-inactivated Fetal Bovine Serum (FBS; Gibco, Waltham, MA, USA), 1% penicillin/streptomycin and 5% CO_2 atmosphere at 37℃.
