# Supplementary material for: Characteristics and pathogenic role of adherent-invasive Escherichia coli in inflammatory bowel disease: Potential impact on clinical outcomes
Source: PLoS One. 2019 Apr 29;14(4):e0216165. doi: 10.1371/journal.pone.0216165 (PMC6488085; doi:10.1371/journal.pone.0216165)
Supplement: S2 Table — (PDF) [file pone.0216165.s002.pdf]

S2 Table. Primers used in this study

Target	Primer	Primer sequence (5' to 3')	Fragment size (bp)	Reference
CEACAM6	Forward	CACAACCTGCCCCAGAATCGTAT	272	[1]
	Reverse	TTGGGCAGCTCCGGGTATACATG		
TNF-α	Forward	CAGAGGGAAGAGTTCCCCAG	325	[2]
	Reverse	CCTTGGTCTGGTAGGAGACG		
IL-17	Forward	AGAGATATCCCTCTGTGATC	520	[3]
	Reverse	TACCCCAAAGTTATCTCAGG		
IL-8	Forward	ATGACTTCCAAGCTGGCCGTGCT	289	[4]
	Reverse	TCTCAGCCCTCTTCAAAAACTTCTC		
COX-2	Forward	CCCTTGGGTGTCAAAGGTAA	169	[5]
	Reverse	GCCCTCGCTTATGATCTGTC		
GAPDH	Forward	TTGGTATCGTGGAAGGACTCA	270	[6]
	Reverse	TGTCATCATATTTGGCAGGTT		

bp, base pairs; CEACAM6, carcinoembryonic antigen-related cell-adhesion molecule 6; TNF-α, tumor necrosis factor alpha; IL, interleukin; COX-2, cyclooxygenase 2; GAPDH, glyceraldehyde 3-phosphate dehydrogenase

All annealing temperatures were 55℃.

References

1. Glas J, Seiderer J, Fries C, Tillack C, Pfennig S, Weidinger M, et al. CEACAM6 gene variants in inflammatory bowel disease. PloS one. 2011;6(4):e19319. Epub 2011/05/12. doi: 10.1371/journal.pone.0019319. PubMed PMID: 21559399; PubMed Central PMCID: PMC3084820.

2. Dharmani P, Strauss J, Ambrose C, Allen-Vercoe E, Chadee K. Fusobacterium nucleatum infection of colonic cells stimulates MUC2 mucin and tumor necrosis factor alpha. Infection and immunity. 2011;79(7):2597-607. Epub 2011/05/04. doi: 10.1128/IAI.05118-11. PubMed PMID: 21536792; PubMed Central PMCID: PMC3191979.

3. Xu X, Wang R, Su Q, Huang H, Zhou P, Luan J, et al. Expression of Th1- Th2- and Th17-associated cytokines in laryngeal carcinoma. Oncology letters. 2016;12(3):1941-8. Epub 2016/09/03. doi: 10.3892/ol.2016.4854. PubMed PMID: 27588143; PubMed Central PMCID: PMC4998098.

4. Singh RK, Lokeshwar BL. Depletion of intrinsic expression of Interleukin-8 in prostate cancer cells causes cell cycle arrest, spontaneous apoptosis and increases the efficacy of chemotherapeutic drugs. Molecular cancer. 2009;8:57. Epub 2009/08/04. doi: 10.1186/1476-4598-8-57. PubMed PMID: 19646263; PubMed Central PMCID: PMC2729725.

5. Hou Z, Falcone DJ, Subbaramaiah K, Dannenberg AJ. Macrophages induce COX-2 expression in breast cancer cells: role of IL-1beta autoamplification. Carcinogenesis. 2011;32(5):695-702. Epub 2011/02/12. doi: 10.1093/carcin/bgr027. PubMed PMID: 21310944; PubMed Central PMCID: PMC3086701.

6. Yamamoto H, Miyoshi N, Mimori K, Hitora T, Tokuoka M, Fujino S, et al. MACC1 expression levels as a novel prognostic marker for colorectal cancer. Oncology letters. 2014;8(5):2305-9. Epub 2014/10/09. doi: 10.3892/ol.2014.2460. PubMed PMID: 25295116; PubMed Central PMCID: PMC4186624.
